# Fingertip replantation (zone I) without venous anastomosis: clinical experience and outcome analysis

**DOI:** 10.1186/s40064-016-3394-8

**Published:** 2016-10-21

**Authors:** An-shi Huan, Subhash Regmi, Jia-xiang Gu, Hong-jun Liu, Wen-zhong Zhang

**Affiliations:** 1 Department of Hand and Foot Surgery, Subei People’s Hospital, Clinical Medical College of Yangzhou University, Yangzhou City, 225001 Jiangsu Province People’s Republic of China; 2 College of Medicine, Yangzhou University, 11th Huaihai Road, Yangzhou City, 225009 Jiangsu Province People’s Republic of China

**Keywords:** Fingertip amputation, Tamai zone I, Artery-only anastomosis, Replantation

## Abstract

**Purpose:**

The purpose of this study was to report our experience of fingertip replantation without venous anastomosis using alternate method to counter post-operative venous congestion.

**Methods:**

30 Patients (18 men and 12 women) with 30 fingertip amputations (Tamai zone I) were treated with artery-only anastomosis fingertip replantation between March 2010 and July 2014. Postoperative venous outflow was maintained by allowing bleeding through wound gaps combined with topical (12500^u^:250mlNS) and systemic (4000 IU SC once daily) heparin. The outcomes of replantation were evaluated using standard evaluating systems.

**Results:**

The average duration of hospital stay was 10 days (range 7–14 days). Twenty-eight (93 %) replanted fingertips survived. Five replanted fingertip experienced postoperative vascular crisis. The estimated post-operative blood loss was about 200–450 ml (mean, 292 ml). Follow-up period ranged from 12 to 24 months (average, 18 months). At final follow-up examinations, the average value of static two point discrimination test was 5.6 mm (range 3–9 mm) and Semmes–Weinstein monofilament test was 3.35 g (range 2.83–4.56 g). The mean range of motion of distal interphalangeal joint was 65.2° (range 0–90°) and all patients returned to their work within 7–18 weeks (average, 11 weeks).

**Conclusion:**

Artery-only fingertip replantation can provide satisfactory cosmetic and functional results. Adequate venous outflow can be obtained by allowing minimal external bleeding through wound gaps combined with topical and systemic heparin.

## Background

Tamai (1982) zone I (distal to the nail base) replantation possess considerable challenge to hand surgeons, because venous anastomosis is extremely difficult, and venous congestion is a leading cause of failure (Barbary et al. 2013; Hattori et al. 2003). Despite having some limitations, artery-only replantation for zone I amputations has gained popularity over the years (Han et al. 2002; Streit et al. 2014; Kim et al. 2014; Whitaker et al. 2012; Chen et al. 2013, 2014; Jeon et al. 2016; Matsuda et al. 1993). Many authors have described various techniques, including external bleeding protocol using pulpar incision (Hasuo et al. 2009), partial nail plate removal (Yabe et al. 2001), and paraungual area stab incision (Gordon et al. 1985) and application of medicinal leech (Han et al. 2002) and mechanical leech (Streit et al. 2014) to counter venous congestion. However, none has provided entirely satisfactory results.

Therefore, in this study, we report our experience of 30 artery-only fingertip replantations and describe an alternative technique to manage postoperative venous congestion. In addition, we rigorously evaluate the outcomes of replantation using standard evaluating systems.

## Patients and methods

We reviewed clinical data records of patients who underwent fingertip replantation in our hospital. Patients with Tamai zone I replantations were included for the study. For each included patients, the following data were recorded: age, sex, mechanism of injury, location of the amputation, ischemia time, operation time, estimated post-operative blood loss, complications and duration of hospital stay and follow-up.

This study was approved by local ethical committee of Subei People’s Hospital and institutional review board of Yangzhou University.

### Surgical procedure

Preoperatively, patients were explained thoroughly about the risks and benefits of the procedure and written informed consents (for both the procedure and inclusion in the study) were obtained. Operation was done under digital nerve block anesthesia with rubber tourniquet (applied at the base of the finger) control.

Under surgical microscope (25× magnification), the debridement of the distal amputated stump and proximal stump was done to isolate vessels and nerves for anastomosis. Fracture fixation was done with K-wire using retrograde trans-fixation method in all cases. Arterial anastomosis was done in an end-to-end fashion in all the cases using non-absorbable microscopic sutures (10-0 or 11-0 prolene sutures). Of 30 fingertip replantations, 24 underwent one artery anastomosis and 6 underwent two artery anastomoses. Venous anastomosis was not possible in all cases. Nerve repair was done in 16 fingers. The nail bed was repaired carefully, using 5-0 absorbable sutures. The skin was loosely sutured using 4-0 prolene sutures, four to six stitches were applied at about 1 cm apart.

### Postoperative regime

All patients were treated with intravenous papaverine (30 mg every 8 h) and subcutaneous heparin (4000 IU once-daily) injections for 2–4 days. Postoperative bleeding was allowed for 12–24 h through wound gaps, and the area was frequently washed with heparinized normal saline solution (12500^u^:250 ml). Replanted fingertips were frequently monitored for vascular crisis and evidences of venous congestion. Patients were kept under controlled temperature of 20–25 °C.

### Follow up outcome evaluation

Patients were followed up regularly. At final follow-up visit, the sensibility outcomes were evaluated using static two point discrimination (s2PD) (using calipers) and Semmes–Weinstein monofilament (SWM) tests (using monofilaments of size 2.83, 3.61 and 4.56 g). In addition, range of motion of DIP joints (degrees) and return-to-work time (weeks) were noted. All the examinations were done by surgeon who was not the part of initial treatment process.

## Results

We enrolled 30 patients (18 men and 12 women), who underwent artery-only fingertip replantation between March 2010 and July 2014. The average age was 34 years (range 19–52 years). The mechanisms of injury were clean-cut injury (13 patients), crush-cut injury (13 patients) and crush-avulsion injury (4 patients). Right hand and left hand ratio was 1:1. The digits involved were thumbs (2 patients), index fingers (10 patients), long fingers (10 patients), ring fingers (6 patients) and little fingers (2 patients). All fingertip amputations were Tamai zone I amputations. The mean ischemia time was 3.4 h (range 1.6–5 h). The mean operation time was 2.4 h (range 1.8–3.3 h) (Table 1).

**Table 1:** Demographic data (31 patients with 31 fingertip amputations)

Cases	Age (years)/sex	Mechanism of injury	Injured finger	Ischemia time (h)	Operation time (h)	Estimated blood loss (ml)	Duration of hospital stay (days)	Number of arterial anastomosis	Outcome
1	20/F	Crush-cut	Left thumb	3.5	2.4	240	8	1	Survived
2	23/M	Cut	Right index	2	2	400	9	1	Survived
3	32/M	Cut	Left ring	2.5	1.8	300	12	1	Survived
4	47/M	Crush-cut	Right index	2.2	2.7	240	9	1	Survived
5	18/M	Crush-Avulsion	Left Long	5	3.3	250	7	1	Failed
6	32/F	Cut	Left index	3.7	1.5	220	8	2	Survived
7	41/F	Crush-cut	Right ring	2.5	2.2	300	10	1	Survived
8	36/M	Crush-cut	Left long	2.2	3.1	350	10	1	Survived
9	41/M	Cut	Right ring	2.7	2.5	300	9	1	Survived
10	45/M	Cut	Left index	1.6	2.3	450	10	1	Survived
11	39/F	Crush-cut	Right ring	2.3	2.7	250	10	1	Survived
12	28/M	Crush-cut	Left long	4.1	2.5	250	12	1	Survived
13	47/F	Crush-cut	Left little	2.7	3.3	280	8	1	Survived
14	35/F	Crush-cut	Right ring	3.3	2.7	200	9	1	Survived
15	34/F	Cut	Right index	3.5	2	320	9	2	Survived
16	25/M	Cut	Left Long	4.5	2.1	220	8	2	Survived
17	52/M	Cut	Right index	3.7	2.6	200	10	1	Survived
18	30/M	Crush-cut	Right long	3.9	2.3	350	7	1	Failed
19	26/M	Crush-Avulsion	Left ring	4.5	2.5	200	10	1	Survived
20	25/F	Crush-cut	Right long	3.2	2.7	220	8	1	Survived
21	34/M	Crush-cut	Right index	4.6	2.3	340	10	1	Survived
22	19/M	Cut	Left Long	3.5	2.2	300	12	2	Survived
23	25/M	Crush-cut	Left index	4.3	2.7	450	14	1	Survived
24	28/M	Crush-Avulsion	Right little	3.7	3	250	8	1	Survived
25	44/F	Cut	Right long	3.5	1.9	320	10	2	Survived
26	37/M	Crush-Avulsion	Left long	4.5	3.2	350	7	1	Survived
27	43/F	Cut	Right long	2.6	2.1	280	9	2	Survived
28	42/F	Crush-cut	Left index	3.5	2.3	240	9	1	Survived
29	34/M	Cut	Right index	3.5	1.8	280	8	1	Survived
30	25/F	Cut	Left thumb	3.4	2	420	9	1	Survived
Mean	33.6			3.4	2.4	292.3	9.3		

*M* male, *F* female

The average duration of hospital stay was 10 days (range 7–14 days). Twenty-eight (93 %) replanted fingertips survived. Five replanted fingertip experienced postoperative vascular crisis, three of them survived after thrombectomy and re-anastomosis. The estimated post-operative blood loss was about 200–450 ml (mean, 292 ml). All patients with survived fingertip were available for follow-up. Follow-up period ranged from 12 to 24 months (average, 18 months). At final follow-up examinations, the average value of static two point discrimination (2PD) test was 5.6 mm (range 3–9 mm) and Semmes–Weinstein monofilament (SWM) test was 3.35 g (range 2.83–4.56 g). The mean range of motion (ROM) of distal interphalangeal joint (DIPJ) was 65.2° (range 0–90°) and all patients returned to their work within 7–18 weeks (average, 11 weeks) (Table 2).

**Table 2:** Follow-up evaluation

Case	Follow-up (months)	2PD (mm)	SWM (g)	ROM of DIPJ (°)	RTW (week)
1	12	5	2.83	80	12
2	12	7	3.61	60	13
3	18	5	2.83	82	12
4	18	5	2.83	80	10
5	12	6	3.61	65	12
6	18	5	2.83	80	8
7	12	7	3.61	70	9
8	18	5	2.83	74	8
9	24	4	2.83	82	12
10	24	3	2.83	90	12
11	12	6	3.61	66	8
12	24	3	2.83	90	10
13	12	6	3.61	55	10
14	18	5	3.61	72	12
15	18	6	3.61	70	10
16	12	9	4.56	0	16
17	15	8	4.56	0	18
18	18	6	3.61	40	10
19	18	6	3.61	70	8
20	18	5	2.83	68	7
21	24	4	2.83	90	12
22	12	7	3.61	40	12
23	24	4	2.83	90	8
24	18	9	4.56	0	14
25	18	7	3.61	60	11
26	24	3	2.83	90	8
27	24	4	2.83	90	9
28	18	6	3.61	72	10
Mean	17.7	5.6	3.35	65.2	10.8

*2 PD* two point discrimination test, *SWM* Semmes–Weinstein monofilament test, *ROM of DIPJ* (°) range of motion of distal interphalangeal joint, *RTW* Return time to work

Complications include complete necrosis (7 %), post-operative vascular crisis (17 %), pulp atrophy (20 %), mild-to-moderate cold intolerance (20 %), nail deformity (20 %), bony mal-union (17 %), joint stiffness (10 %), and neuroma formation (7 %) (Table 3).

**Table 3:** Prevalence of complications

Complications	Number of cases
1. Complete necrosis	2 (7 %)
2. Vascular crisis	5 (17 %)
3. Mild to moderate cold intolerence	6 (20 %)
4. Pulp atrophy	6 (20 %)
5. Joint stiffness	3 (10 %)
6. Bony mal-union	5 (17 %)
7. Nail deformitiy	6 (20 %)
8. Neuroma formation	2 (7 %)

## Discussion

Fingertip amputations are very common in developing world. There are varieties of treatment options available, such as replantation, revision amputation, composite grafts, local flaps, and free tissue transfer (Barbary et al. 2013; Peterson et al. 2014). However, the ideal reconstruction must restore digital length and provide adequate sensation and satisfactory range of motion (Peterson et al. 2014). Therefore, successful replantation is always superior to any other methods of reconstruction (Yabe et al. 2012).

**Fig. 1:**
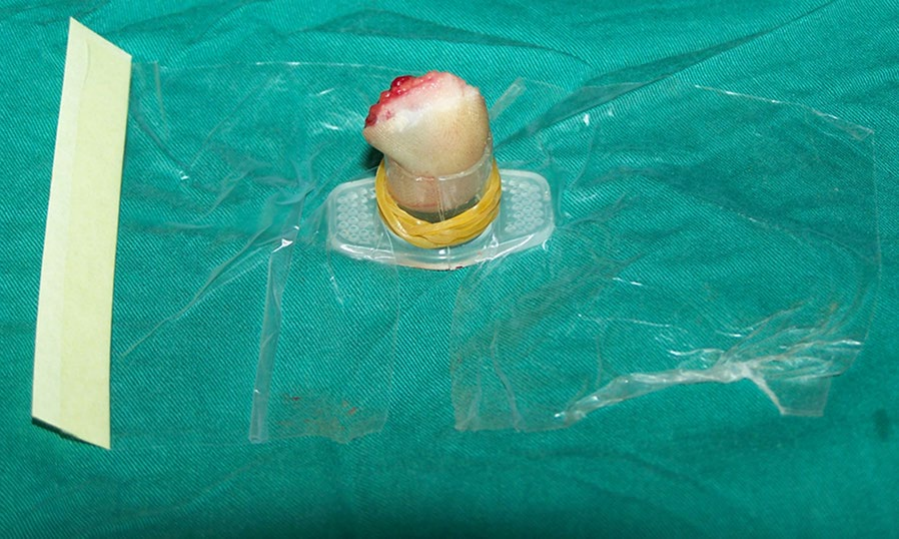
1/5th portion of the 5 cc syringe barrel was split from one side and the amputated part of finger tip was placed inside (injured portion facing upward). The elastic rubber band was used to wrap the barrel and fix the amputated part. The amputated part of fingertip along with the syringe barrel was dipped into disinfectant solution and then placed on surgical table

**Fig. 2:**
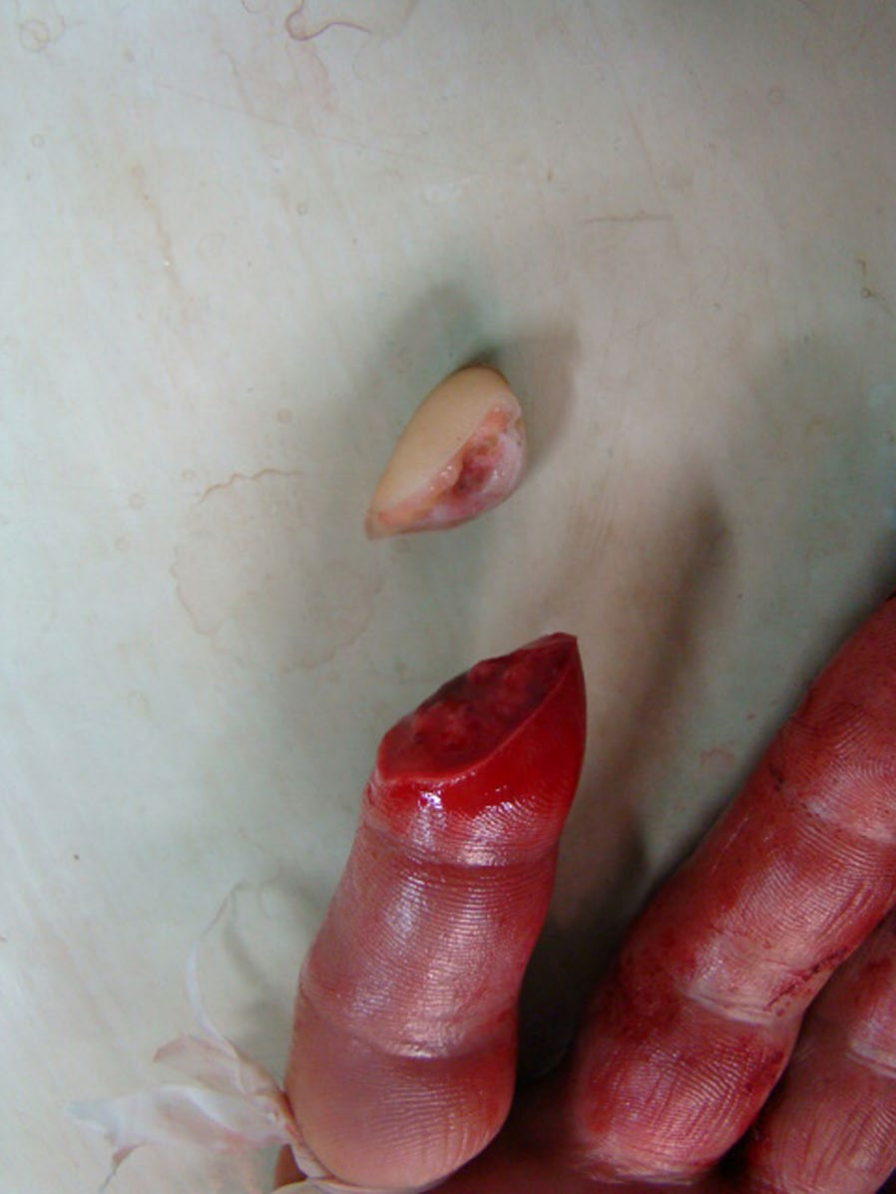
A 23 year old male presented with right index fingertip amputation (Tamai zone I)

**Fig. 3:**
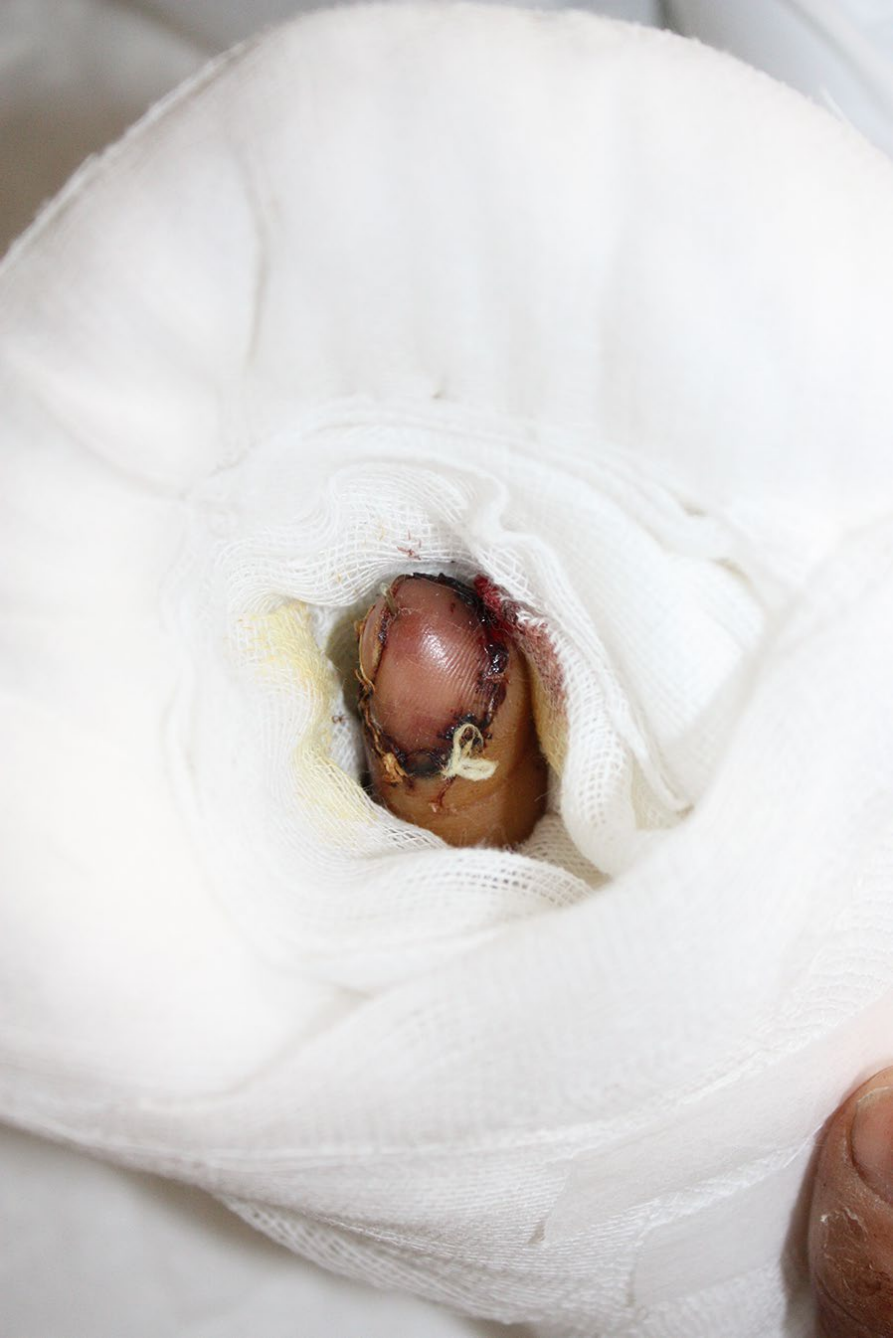
Fingertip replantation was done using artery-only anastomosis technique. Postoperative bleeding was allowed through the wound gaps

**Fig. 4:**
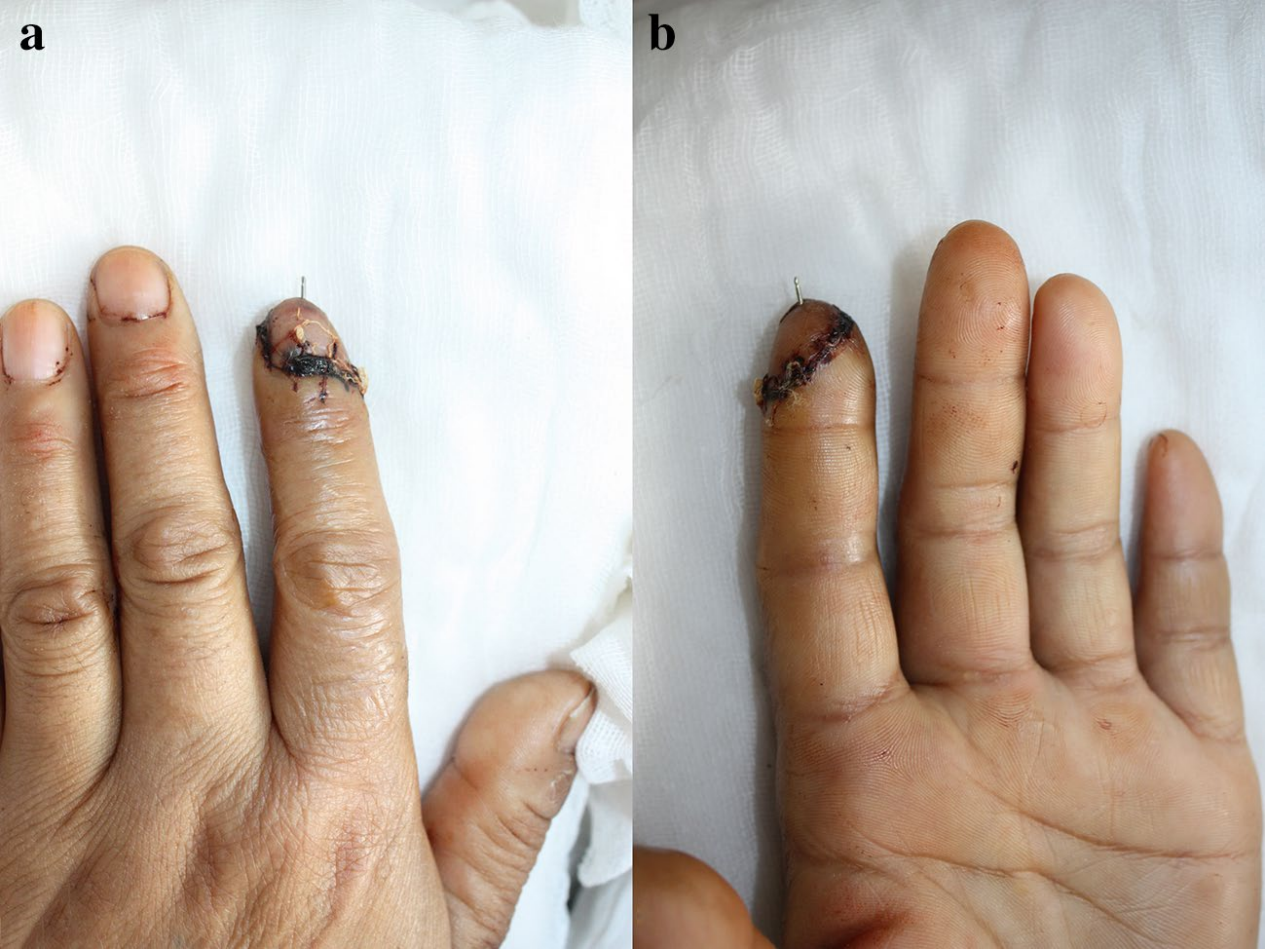
Replanted fingertip at the time of discharge: dorsal view (**a**) and palmar view (**b**)

**Fig. 5:**
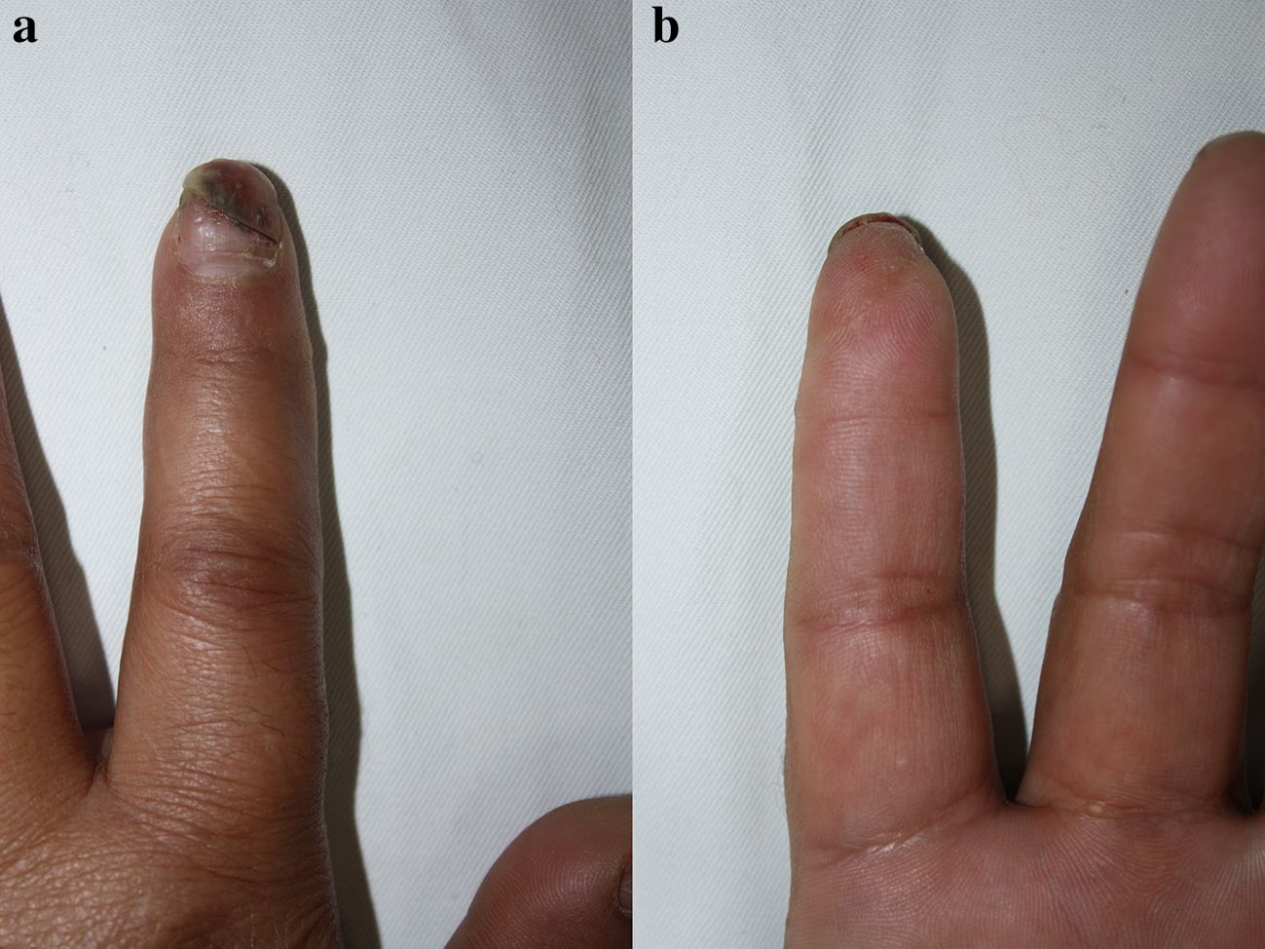
Replanted fingertip at 12 months: dorsal view (**a**) and palmer view (**b**)

Despite being an ideal choice of reconstruction, fingertip replantation is not commonly performed because of some inherent difficulties, including identification of blood vessel, small vessel anastomosis and post-operative venous congestion (Kim et al. 2013). In our study, we used a different method to fix the amputated stump during debridement to facilitate the identification process (Fig. 1). This method allows better access and avoids further (iatrogenic) damage to neurovascular structures. Micro-vascular anastomosis has become easier these days with the availability of surgical microscopes (25× magnifications). However, extreme care should be taken during the procedure to avoid endothelial injury, which is the main cause for post-operative vascular crisis (Figs. 2, 3, 4, 5).

Venous congestion after artery-only fingertip replantation (zone I) is an inevitable phenomenon (Hattori et al. 2003). If not addressed early, it becomes problematic and may cause failure (Buntic and Brooks 2010; Venkatramani and Sabapathy 2011). Adequate restoration of venous outflow is required to obtain success. Various techniques, such as pulpar incision, partial nail plate removal, and paraungual area stab incision have been used to allow post-operative bleeding to maintain adequate venous outflow (Hasuo et al. 2009; Yabe et al. 2001; Gordon et al. 1985). In addition, some other techniques, such as application medicinal leech and mechanical leech have also been used (Han et al. 2002; Streit et al. 2014). These techniques have been extremely successful. However, bleeding is often very profound and may require blood transfusion. Erken et al. (2013) reported controlled nail bed bleeding protocol for artery-only fingertip replantation and 15 of 22 patients required blood transfusion.

Arteriovenous (AV) shunting is another alternative to restore adequate venous drainage (Nichter et al. 1985; Chen et al. 2005). This technique is commonly performed as a salvage procedure for arterial inflow or venous drainage when the standard artery-to-artery or vein-to-vein anastomoses become impossible. Nitcher et al. (1985) performed an experimental study which strongly supports efferent AV shunting (single arterial inflow with efferent AV fistula for venous outflow) technique in the management of replants with absent venous drainage. However, this technique requires patent venous structures at the amputated stump. In addition, literature lacks enough evidence to support the usage of this technique (efferent AV shunting) to restore venous outflow in artery-only fingertip replantation. Peterson et al. (2014), in their recent study, have concluded that artery-only fingertip replantation (zone I) may not require obligatory external bleeding to restore venous outflow. We also support his findings and agree to the fact that venous outflow could be managed by the bleeding that occurred from wound-edge and bone marrow reflux (Tanaka et al. 1998; Chen et al. 1991). Therefore, in our study we sutured skin loosely and allowed post-operative bleeding through the suture gaps. We applied heparinized normal saline solution (12500^u^:250 ml) topically to avoid obstruction due to clot formation. We observed minimal oozing of blood for about 12–24 h. Mild venous congestion was observed in all patients, which resolved in 2–4 days. Fingertip temperature and blood color on pinprick were used as the indicators to determine successful replantation. Total estimated post-operative blood loss was about 200–450 ml and no blood transfusion was required.

Despite having successful results, we do not recommend intended artery-only anastomosis in Tamai zone I replantations. Surgeons should make every effort to carefully isolate the vein and perform venous anastomosis. In our study, we were able to isolate veins only in patients who presented with clean-cut amputations. However, those veins were not suitable for anastomosis. In addition, we performed two arterial anastomoses in 6 patients and found that venous congestion subsided early (2 days) in those patients. This proves the fact that better post-operative perfusion decreases the duration of venous congestion and improves the survival (Matsuda et al. 1993).

In our study, postoperative vascular crisis was observed in 5 replanted fingertips. Three replanted fingertip survived after thrombectomy and re-anastomosis whereas ischaemic necrosis occurred in two cases. Patients with survived fingertips (n = 28) were included for final follow-up evaluation. According to the results, sensibility outcomes (mean s2PD = 5.6 mm and SWM = 3.35 g) were satisfactory. The sensibility outcomes tend to improve with time (at 24 month follow up, s2PD = 3 mm). There was no significant difference (P = 0.35, α = 0.05, using ANOVA) in s2PD outcomes between patients with nerve anastomosis (mean ± S.D. = 5.5 ± 1.78 mm, n = 16) and without nerve anastomosis (mean ± S.D. = 6.07 ± 1.49 mm, n = 14). Our outcomes could not deny the fact that fingertip replantation can provide satisfactory sensory recovery without nerve anastomosis (Ozcelik et al. 2008). However, some complications, such as pulp atrophy and neuroma formation were found in patients in whom nerve anastomosis were not performed. Therefore, we believe the importance of nerve anastomosis should not be neglected and surgeons should perform nerve repair whenever possible.

In our study, the ROM of DIPJ was satisfactory and all patients returned to their normal work. Our results were better compared to that reported in the literature (Sebastin and Chung 2011). However, there were some associated complications, such as cold intolerance, pulp atrophy, bony malunion, joint stiffness, and neuroma formation.

Our results showed that fingertip replantation is superior to any other method of reconstruction for the treatment of fingertip amputation. However, the reliability of other methods of reconstruction should not be neglected (Wang et al. 2013). Moreover, the choice of technique should depend upon patients’ overall physical and socio-economic conditions, surgeon’s microsurgical skills and availability of high facility centers.

In conclusion, artery-only fingertip replantation can provide satisfactory cosmetic and functional results. Adequate venous outflow can be obtained by allowing minimal external bleeding through wound gaps combined with topical and systemic heparin.
